# Information Provision for Patients Who Undergo Resection of Colorectal Liver Metastases

**DOI:** 10.1007/s13187-025-02573-7

**Published:** 2025-02-01

**Authors:** Nazim Bhimani, Mbathio Dieng, Patrick J. Kelly, Thomas J. Hugh

**Affiliations:** 1 Upper Gastrointestinal Surgical Unit, Royal North Shore Hospital, St Leonards, NSW Australia; 2 Faculty of Medicine and Health, University of Sydney, Sydney, NSW Australia; 3 Sydney School of Public Health, University of Sydney, Sydney, NSW Australia; 4 Northern Clinical School, University of Sydney, Sydney, NSW Australia

**Keywords:** Information provision, Colorectal liver metastases, Patient satisfaction, Cancer survivors

## Abstract

Information provision to patients is helpful prior to and during cancer treatment. However, the level of information required or warranted varies, and no studies have examined this in patients who have had resection of colorectal liver metastases (CRLM). The aims of this study are to assess how patients perceive information received from different healthcare providers and to examine factors that might be associated with the quality and satisfaction of the information received. This was a cross-sectional study of patients with a potentially curative resection for CRLM between 2010 and June 2021. Patients completed the European Organisation for Research and Treatment of Cancer QLQ-INFO25 questionnaire. In total, 121 patients underwent resection. Of these, 85 were alive and were sent the questionnaire, and 52 (61%) responded. Overall, patients were very satisfied with the information they received and found it helpful (median score 100). No specific patient or disease-related factors were associated with the degree of satisfaction. This study demonstrates excellent overall satisfaction with the information provided to patients with CRLM. Areas of improvement include information about other supportive services outside the hospital. This information may be given by the specialists providing the care but may also be provided by the patient’s general practitioners and the cancer nurse coordinators.

## Introduction

Information provision involves health workers providing cancer-related information to patients in either oral, written or another form [1]. This information may include the cause of a disease, how it is diagnosed, the purpose of any medical tests performed, the treatment options available (including side effects), the prognosis of the disease, and any support services available. In cancer care, this is particularly worthwhile for both patients and their families because it may assist in making informed decisions about their disease and treatment options. A detailed understanding of the issues may also improve adherence to treatment recommendations, may reduce stress levels, and assist with developing a financial plan to deal with any possible out-of-pocket expenses or loss of income because of treatment. Many patients who receive detailed information soon after their diagnosis report improved overall satisfaction and an increased sense of control over the diagnosis and treatment [1, 2]. Unfortunately, many other cancer patients report that they did not receive adequate information [3, 4]. This is likely due to a variety of reasons, sometimes because of a lack of understanding of what patients and healthcare providers deem appropriate or because of ineffective communication strategies. Furthermore, there may be some conscious or unconscious resistance to receiving medical information because it is complex and can be emotionally distressing to hear and process [5, 6]. From the published literature to date, it seems that most cancer patients want as much information as possible about their diagnosis, treatment options, survival, the financial implications, the likely rehabilitation, and any available support services. However, the extent and type of information provided may vary by the age, gender, education level, and levels of emotional distress [7–9].

Patients with colorectal liver metastases (CRLM) may be overwhelmed by the gravity of having an advanced cancer diagnosis, the difficulty in understanding the various treatment options, or the potential immediate and long-term cost implications of their disease. Stress levels will likely depend on whether the proposed cancer treatment is potentially curable or palliative only [4]. Regardless, and depending on the clinical situation and the patient’s wishes, it is important for healthcare workers not to burden patients with excessive information. Clinicians need to make a judgement about what information they feel their patient wants or needs to know.

For those being considered for liver resection, there are numerous resources available beyond the information provided by the immediate healthcare providers. However, these may not all be necessary or helpful. In Australia, online resources providing background information and details of support services related to the diagnosis and treatment of CRLM include the Cancer Council of Australia, Cancer Australia, the Clinical Oncology Society of Australia, the Australian Cancer Trials Group, the Gastroenterological Society of Australia, and The Australian Liver Foundation. Apart from the variability in the content and relevance available, there may also be variability in the uptake of this information because some patients may not want to know too much or may not have easy access to the internet.

To date, there have been no studies from Australia assessing the type or quality of information provided to patients who undergo resection of CRLM. The aim of this study was to use the European Organisation for Research and Treatment of Cancer (EORTC) QLQ-INFO25 questionnaire to explore how patients perceived the information they received from the different healthcare providers [10]. This was done retrospectively in a cohort of patients who had potentially curative resection of CRLM. A detailed analysis was undertaken to assess what factors might have influenced the perception of information provision.

## Materials and Methods

A single-unit cross-sectional study of patients who underwent a potentially curative resection for CRLM between 2010 and June 2021 at two hospitals (Royal North Shore Hospital and North Shore Private Hospital) was undertaken. Ethics approval was given by the Northern Sydney Local Health District Human Research Ethics Committee (LNR/11/HAWKE/258). Patients who had at least one year of follow-up were retrospectively identified from a prospectively collected database. Those who underwent resection prior to 2010 were not included as the modern era of treating CRLM includes the use of biological agents (cetuximab and bevacizumab), and these treatments were not available or were not routinely used prior to this time. Patients were contacted over the telephone, and after agreeing to participate, they were then posted the questionnaire. Those who did not return the questionnaire after three months were contacted again and encouraged to participate.

### Questionnaire

The EORTC QLQ-INFO25 questionnaire was administered. This is a 25-item cancer-specific instrument consisting of four scales to assess information about the disease, medical tests, treatment, and other services, with eight single-item scales to assess information about other areas and satisfaction with the information provided. Scores were determined using the EORTC manual [11]. All scores range from 0 to 100, where a score of 100 represents a high level of information, and a score of 0 represents a low level of information.

### Patient Population

Information obtained from the database included demographic data (age, sex, marital status, employment status, education level, and household income), pre-existing medical conditions (Charlson Comorbidity Index (CCI) and American Society of Anaesthesiologists (ASA)), radiological investigations (timing and primary sidedness), pre-operative treatments (chemotherapy + / − biological agents, radiotherapy, trans-arterial chemoembolisation (TACE), selective internal radiation therapy (SIRT), live ablation, or portal vein embolisation), intra-operative details (operative technique, extent of liver resection, resection margin, and tumour differentiation), post-operative outcomes (complications, length of stay, and recurrence), and post-operative treatment (chemotherapy + / − biological agents, liver ablation, and locoregional therapy). Neoadjuvant chemotherapy and biological agents were defined as treatment provided six months before the liver resection. The extent of the liver resection was defined as either major or minor, where a major liver resection involved the removal of at least three contiguous Couinaud segments.

### Statistics

Categorical variables were presented as counts and percentages. Continuous variables were expressed as a mean and standard deviation for normally distributed data and analysed using two-tailed *T*-tests. Continuous variables not normally distributed were expressed as a median and range and analysed using the Mann–Whitney *U* test. All statistical analyses were performed using Stata® BE for Windows® version 17.1 (StataCorp, College Station, Texas, USA).

## Results

**Fig. 1:**
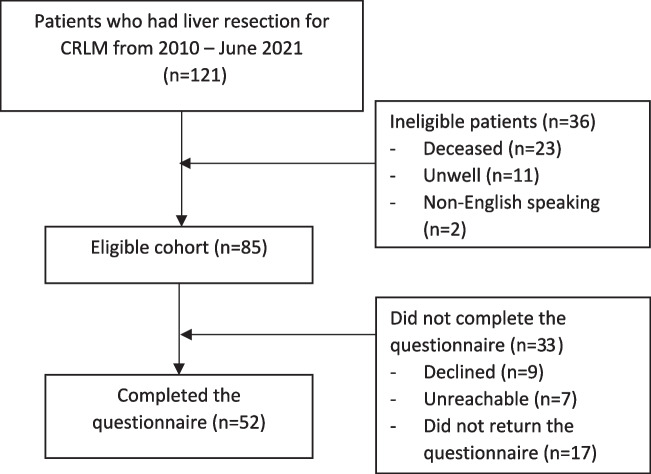
Flow chart of patients included in the study

One hundred and twenty-one patients had a liver resection for CRLM during the period January 2010 to June 2021. Of these, 47 patients (39%) achieved long-term survival (> five years), 23 patients (19%) were deceased, 11 patients (9%) were too unwell to complete the questionnaire, and 2 patients (2%) were deemed unsuitable for the study as they were unable to speak English. Eighty-five patients were posted the questionnaire, although nine patients declined to complete the questionnaire for an unspecified reason, seven patients were unreachable after three attempts at contact, and 17 patients did not return the questionnaire and were therefore excluded. The final cohort included 52 respondents (overall 61% response rate), and these are the subject of this study (Fig. 1).

Demographic characteristics are shown in Table 1. Most survey participants were men (60%), and the median age at the time of completion of the survey was 69 years. Most were either married or living with a partner (71%), were retired (60%), and had at least completed high school (85%). When the patient was the primary earner in the household, 39% were earning ≥ AUS80,000 per year. In the study cohort, 67% of patients had private health insurance, and the median time at which the survey was completed following the CRLM resection was 4.0 years (range 1.0–13.1 years).

**Table 1:** Demographic characteristics

**Variable**	
Gender, *n* (%)	
Male	31 (60)
Female	21 (40)
Median age when survey was completed (range)	69 (33–85)
American Society of Anaesthesiologists, *n* (%)	
1	32 (61)
2	18 (35)
3	2 (4)
Median Charlson Comorbidity Index (range)	8 (6–12)
Marital status, *n* (%)	
Single	4 (8)
Married or living as a married couple	37 (71)
Separated	3 (5)
Divorced	4 (8)
Widowed	4 (8)
Employment status, *n* (%)	
Full-time work	14 (27)
Part-time work	6 (11)
Seeking work	1 (2)
Retired	31 (60)
Education level, *n* (%)	
Primary	1 (2)
Some high school	7 (13)
Completed high school	16 (31)
Vocational education training	12 (23)
Undergraduate university	12 (23)
Postgraduate university	4 (8)
Annual household income, *n* (%)	
Less than $19,999	5 (9)
$20,000 to $39,999	12 (23)
$40,000 to $59,999	6 (12)
$60,000 to $79,999	9 (17)
$80,000 to $99,999	3 (5)
$100,000 to $119,999	6 (12)
$120,000 to $139,999	1 (2)
$140,000 to $159,999	2 (4)
$160,000 to $179,999	2 (4)
Above $200,000	6 (12)
Main earner in the household, *n* (%)	
Patient with cancer	23 (44)
Spouse	10 (19)
Shared between survivor/spouse	19 (37)
Private health Insurance, *n* (%)	
Yes	35 (67)
No	17 (33)
Adults in household, *n* (%)	
1	10 (19)
2	33 (64)
3 or more	9 (17)
Children in household, *n* (%)	
0	41 (79)
1 or 2	11 (21)
Median years of survey completion after surgery	4.0 (0.8–13.1)

Table 2 describes the tumour characteristics and treatment received. Most patients had their primary tumours removed first (83%), were diagnosed with a Dukes C primary tumour (61%), had a left-sided tumour (83%), presented with synchronous disease (56%), received neoadjuvant chemotherapy (83%), had a minor liver resection (77%), did not have a post-operative complication (61%), had an R0 liver resection (83%), received post-operative chemotherapy (73%), and did not develop recurrent disease (60%).

**Table 2:** Tumour biology and treatment received

**Variable**	
**Primary resection details**	
Order of resection	
Primary first	43 (83)
Liver first	2 (4)
Combined	7 (13)
Dukes stage	
Dukes A	3 (6)
Dukes B	17 (33)
Dukes C	32 (61)
Primary site	
Colon	31 (60)
Rectum	21 (40)
Sidedness	
Left	43 (83)
Right	9 (17)
Timing of liver metastases	
Metachronous	23 (44)
Synchronous	29 (56)
**Treatment prior to liver resection**	
Neoadjuvant chemotherapy	43 (83)
SBRT OR Chemoradiotherapy	4 (8)
TACE	0 (0)
SIRT	0 (0)
RFA or MVA	0 (0)
Portal vein embolisation	1 (2)
**Operative details (*n* = 61)**	
Operative technique	
Open	51 (84)
Laparoscopic	7 (11)
Laparoscopic converted to open	3 (5)
Liver resection	
Major	14 (23)
Minor	47 (77)
Median length of stay in days (range)	7 (3–30)
Complications	24 (39)
Tumour differentiation	
Well	5 (8)
Moderate	30 (51)
Poor	1 (2)
Not stated	23 (39)
**Treatment and outcomes following liver resection(s) (*n* = 52)**	
Chemotherapy	38 (73)
SBRT	8 (15)
Chemoembolisation/TACE	1 (2)
SIRT	1 (2)
RFA or MVA	2 (4)
**Recurrence**	21 (40)

*SBRT* stereotactic body radiotherapy, *TACE* trans-arterial chemoembolisation, *SIRT* selective internal radiation therapy, *RFA* radiofrequency ablation, *MWA* microwave ablation

Table 3 summarises the outcomes of the EORTC QLQ-INFO25 questionnaire. The responses provided by patients were in relation to all healthcare providers during the treatment for CRLM. Overall, patients were satisfied with the information they received, and most found it helpful, with a median score of 100 in both categories. However, patients only infrequently received written information (median score of 0), although many who had chemotherapy received audio-visual information on either a CD or DVD about this treatment, with a median score of 100. Only six patients (12%) indicated they requested more information during their treatment, although it was not stated which clinician they were referring to. Specific examples of inquiries included asking for more information about: the management and identification of symptoms, the treatment options for metastatic disease, a detailed explanation of their scan results, information in relation to support groups, including psychological support and financial planning, and written information that they might provide to friends and family members to avoid repeating themselves. One patient did not specify what information they wanted.

**Table 3:** Results of the EORTC QLQ-INFO25 questionnaire

Variable	Median (range)
Information about the disease	75 (17–100)
Information about medical tests	100 (33–100)
Information about treatments	78 (17–100)
Information about other services	50 (0–100)
Information about different places of care	33 (0–100)
Information about things you can do to help yourself	67 (0–100)
Did you receive written information?	0 (0–100)
Did you receive Information on CD/video	100 (0–100)
Satisfaction with the information received	100 (0–100)
Wish to receive more information	100 (0–100)
Wish you had received less information	100 (0–100)
Overall, the information has been helpful	100 (33–100)
Global Score	74 (31–100)

Overall, patients responded positively to the information about their medical tests (the purpose, what was involved, and the results), with a median score of 100 in this domain. Patients were also mostly satisfied with the information they received about their disease (diagnosis, spread, cause, and control of disease), with a median score of 75, and information about their treatment (treatment received, benefit, side effects, effect of treatment on the disease, social and family life, and sexual activity), with a median score of 78. Based on the data obtained from this survey, there were several areas that could be improved. These included information about other services (help outside the hospital, rehabilitation services, and psychological support), with a median score of only 50; information about different places of care (alternative hospitals, outpatient services, and treatments at home), with a median score of only 33, and information about things patients might do to help themselves (rest, contact with others), with a median score of 67.

Table 4 compares the EORTC QLQ-INFO25 questions relating to satisfaction and helpfulness with patient characteristics and disease factors. Whilst there were differences in the median score in some patient and disease factors, there was no statistical difference. Males, younger patients, patients with fewer comorbidities, and those with no tertiary qualifications scored better than females, older patients, patients with multiple comorbidities, and patients with tertiary qualifications. Whilst not statistically significant, there were some subgroups of patients with a higher median satisfaction score than their counterparts, including those with colon cancer (compared with rectal cancer), those who had synchronous disease (compared with metachronous disease), those who underwent a major liver resection (compared with minor liver resections), those who did not require repeat liver resections (compared with those who did), and those who experienced a complication or received treatment after their liver resection (compared with those who did not).

**Table 4:** Median score and range examining the relationship between EORTC QLQ-INFO25 and patient/disease factors

**Variable**	Satisfaction with the information received	Overall, the information has been helpful
Gender		
Male	67 (0–100)	67 (33–100)
Female	100 (33–100)	100 (33–100)
*p*-value	0.418	0.224
Age		
< 60	100 (33–100)	100 (33–100)
≥ 60	83 (0–100)	100 (33–100)
*p*-value	0.743	0.846
< 70	100 (0–100)	100 (33–100)
≥ 70	67 (0–100)	83 (33–100)
*p*-value	0.361	0.799
American Society of Anaesthesiologists		
1	67 (0–100)	83 (33–100)
2 or 3	100 (0–100)	100 (33–100)
*p*-value	0.501	0.455
Charlson Comorbidity Index		
< 9	100 (0–100)	100 (33–100)
≥ 9	67 (33–100)	67 (67–100)
*p*-value	0.840	0.946
Cohabitation status		
Single	100 (0–100)	67 (33–100)
Couple	100 (0–100)	100 (33–100)
*p*-value	0.940	0.261
Employment status		
Employed	83 (0–100)	100 (33–100)
Non-employed	100 (0–100)	83 (33–100)
*p*-value	0.896	0.640
Education level		
Tertiary educated	67 (0–100)	100 (33–100)
No tertiary education	100 (33–100)	100 (67–100)
*p*-value	0.185	0.486
Annual household income		
< $80,000	100 (0–100)	67 (33–100)
≥ $80,000	67 (0–100)	100 (33–100)
*p*-value	0.725	0.333
Children in household		
0	67 (0–100)	100 (33–100)
1 or 2	100 (33–100)	100 (67–100)
*p*-value	0.409	0.414
Time after surgery		
< 5 years	100 (0–100)	100 (33–100)
≥ 5 years	100 (0–100)	67 (33–100)
*p*-value	0.959	0.795
Primary site		
Colon	100 (0–100)	100 (33–100)
Rectum	67 (33–100)	67 (33–100)
*p*-value	0.615	0.603
Timing of liver metastases		
Metachronous	67 (0–100)	67 (33–100)
Synchronous	100 (33–100)	100 (33–100)
*p*-value	0.206	0.215
Liver resection		
Major	100 (0–100)	100 (33–100)
Minor	83 (0–100)	83 (33–100)
*p*-value	0.774	0.630
Multiple liver resections		
Yes	67 (33–100)	83 (33–100)
No	100 (0–100)	100 (33–100)
*p*-value	0.225	0.535
Complications		
Yes	100 (33–100)	100 (33–100)
No	83 (0–100)	67 (33–100)
*p*-value	0.492	0.213
Any post-liver resection treatment		
Yes	100 (33–100)	100 (33–100)
No	67 (0–100)	67 (33–100)
*p*-value	0.279	0.336
Recurrence		
Yes	100 (33–100)	67 (33–100)
No	67 (0–100)	100 (33–100)
*p*-value	0.726	0.603

## Discussion and Conclusion

### Discussion

This study included a mixture of long-term survivors and patients undergoing treatment for recurrent disease following resection of CRLM. Patients were, in general, very satisfied with the information provided to them during their treatment and they found it helpful. There were no specific factors that influenced patient satisfaction with the information they received or how helpful it was during treatment. Areas of improvement identified include providing information about other supportive services within the hospital (e.g., the cancer nurse coordinator (CNC)) or outside the hospital (psychological support, occupational therapy, physiotherapy, and acupuncture services) and additional support options from in-home support nurses or other allied health professionals.

In a study from Spain on patient satisfaction with information provision in patients with colorectal cancer (CRC), Rodriguez et al. compared the inpatient and outpatient settings [12]. Overall, patients were happy with the information provided, as was found in the current study. However, the provision of information about such things as supportive services outside the hospital or about the causes of the disease were rated poorly by patients. In a similar study from Sweden, which has a universal healthcare system, Lithner et al. examined patients who underwent resection of CRC using the EORTC QLQ-INFO25 questionnaire, and these patients rated their experience with information provision poorly across all the domains [13]. They received the most information about their medical tests and the disease itself, but the least about other supportive services outside the hospital. Interestingly, these authors found that women and patients with significant comorbidities received the least amount of information. Spain and Sweden both have universal healthcare systems funded primarily by tax revenue. It might be expected that services are less fragmented than in other countries with large components of private healthcare. However, this does not appear to guarantee an adequate level of information provision during cancer care. To date, minimal studies have assessed the impact of the type of healthcare system on the provision of information provided to cancer patients. The findings in the study from Spain and Sweden may simply reflect the clinicians’ understanding or bias about whether information provision is helpful or not [1].

Husson et al. conducted two studies in the Netherlands comparing information provision in different cancer types. The first was in patients with either primary or metastatic CRC (mCRC), and the second was in patients with non-GIT cancers (endometrial, Hodgkin lymphoma, non-Hodgkin lymphoma, and myeloma) [14, 15]. In both studies, they found low average scores similar to the findings of Lithner et al. A consistent theme across all studies is that information provision is the lowest in relation to support services available outside the hospital, including receiving treatment at home.

From the literature published to date, it is evident that irrespective of the healthcare system where the patient is being treated, there seems to be inconsistency in the delivery of information to patients, suggesting room for improvement. One way to improve information provision might be for the treating clinician to provide written information on an ongoing basis so that patients can better understand information explained verbally during consultations. This could be in the form of a regular follow up letter to the patient, similar to what is written by a specialist to the general practitioner (GP) following a consultation.

Whilst numerous online resources are available, surgeons or medical oncologists providing a summary or brochure with specific links and resources for further information as a routine part of their care may give the patient more confidence and the opportunity to absorb the information and ask questions. This information may include the cause of the disease, information about the treatment options, and any self-help advice. Ideally, all specialist consultations should be for an adequate amount of time to allow patients the opportunity to ask questions, especially during follow-up consultations or just prior to commencing treatment, where the discussion is recorded and documented in writing, which may reduce concerns and clarify outstanding issues. There is an opportunity for professional colleges (especially the Royal Australasian College of Surgeons and the Royal Australasian College of Physicians) to consider introducing training and workshops for junior doctors on communication, particularly in cancer care. Currently, there are a few dedicated training or continuing professional development opportunities offered in Australia. There are some communications skills training modules offered by Cancer Australia that might also be mandated to improve the provision of information to patients in the future [16].

The provision of information to patients undergoing any form of treatment for CRLM, including liver resection, is a complex issue that transcends the type of healthcare system. Effective information provision can have a significant impact on patient outcomes and beyond just making the patient treatment pathway easier. High levels of information provision reduce levels of anxiety and stress, improve treatment adherence, increase the sense of patient empowerment during treatment, and improve quality of life [17–19].

In this study, information about other services, including rehabilitation and psychological support, were particularly inadequate. It is not clear who should inform patients about these services, at what stage they should be provided, and how patients can best take advantage of them. Undoubtedly, healthcare providers should relay as much information as possible to those in closest contact with the patient, including their GP, family members, or the relevant CNC. A systematic review by Meiklejohn et al. supports a greater role for GPs in care coordination, cancer screening, and the management of the physical and psychological effects of the diagnosis and treatment of cancer [20]. Vogel et al. also studied the role of a GP in cancer care and found that most patients wanted their GP to offer more complementary and alternative medicine options [21].

The limitations of this study include the fact that a survey such as this involves recall bias, particularly in patients diagnosed with CRLM who were treated many years ago, as well as reliance on self-reported measures of information provision. From the data collected, it was not possible to assess if a specific clinician or type of specialist was responsible for either good or bad information provision. Furthermore, the EORTC QLQ-INFO25 assessment tool does not describe when the information was provided to the patient. A future area of work might involve assessing information provision prospectively at different time points, such as at the time of diagnosis, before their primary cancer surgery, before the administration of chemotherapy, and before the liver resection. The cross-sectional nature of the current study provides a snapshot in time, limiting the causal association between the outcomes and the factors of interest. Due to the number of deceased patients and non-responders, the sample size was small. Regardless, this is the first study to assess patient information provision in patients who have undergone liver resection for CRLM.

### Conclusion

This study showed that, overall, patients who underwent treatment for CRLM received sufficient information about their medical tests and proposed treatment, were satisfied with the information they received, and found it helpful. However, some patients felt they could have received more information about additional support services. Information provision can improve, and it is likely that this will only be achieved through further education of specialists, GPs, and allied health staff about the value of high-level communication and by ensuring there is appropriate infrastructure support to ensure this happens.

## Data Availability

The research data includes sensitive and confidential information about patient data; for this reason, data has not been deposited in a data repository. De-identified data may be requested from the corresponding author at a reasonable request.
